# Exploring the role of trifarotene against RAR-α: an investigation of expression pattern and clinicopathological significance of RAR-α in breast cancer

**DOI:** 10.3389/fphar.2024.1361679

**Published:** 2024-06-07

**Authors:** Nusrat Jan, Shazia Sofi, Adel Abo Mansoor, Adil Abdelrahim, Irshad Ahmad, Abdullah Almilabairy, Fuzail Ahmad, Manzoor Ahmad Mir

**Affiliations:** ^1^ Department of Bioresources, School of Biological Sciences, University of Kashmir, Srinagar, India; ^2^ Department of Clinical Laboratory Sciences, College of Applied Medical Sciences (CAMS), King Khalid University, Abha, Saudi Arabia; ^3^ Department of Medical Rehabilitation Sciences, College of Applied Medical Sciences (CAMS), King Khalid University, Abha, Saudi Arabia; ^4^ Department of Family and Community Medicine, Faculty of Medicine, Al Baha University, Al Baha, Saudi Arabia; ^5^ College of Applied Sciences Almaarefa University, Riyadh, Saudi Arabia

**Keywords:** chemoresistance, breast cancer, metastasis, retinoic acid receptor, oncogene, expression pattern

## Abstract

**Introduction:**

The members retinoic acid receptors (RARs) (α, β, and γ) and retinoid X receptors (RXRs) (α, β, and γ) belong to the retinoid receptor family. They regulate the biological action of classical retinoids through nuclear retinoid receptors, a transcription factor that is regulated by ligands. Through the binding of particular retinoic acid-responsive elements (RAREs) located in target gene promoters, RARs and members of the RXRs form heterodimers. By binding to its nuclear receptors and triggering the transcription of the target genes downstream, retinoic acid (RA) mediates the expression of certain genes. Retinoids so mainly control gene expression to carry out their biological actions. RARs are essential for many biological processes, such as development, immunity, reproduction, organogenesis, and homeostasis. Apart from their physiological functions, RARs are also linked to pathologies and tumors due to mutations, protein fusions, changes in expression levels, or abnormal post-translational changes that lead to aberrant functions and homeostasis breakdown. The oncogenic development of animal tissues or cultured cells is linked to altered expression of retinoid receptors. The RAR-α is over-expressed in several malignancies. Increased invasion and migration in several cancer forms, including HNSC carcinoma, pediatric low-grade gliomas, lung adenocarcinoma, and breast cancer, have been linked to its upregulated expression. Numerous approved therapeutic regimens targeting RAR-α have been developed, improving patient survival rates.

**Objective:**

This study’s main objective was to identify novel RAR-α-targeting drugs and evaluate the expression patterns of RAR-α in breast cancer patients.

**Methodology:**

*In-silico* investigation using a variety of bioinformatics tools like UALCAN, TISCH, TIMER 2.0, ENRICHR, and others were employed to examine the expression of RAR-α. Further we evaluated *in-silico* inhibition of RAR-α with trifarotene and also tested the cytotoxicity of trifarotene in breast cancer cells.

**Results:**

Our research indicates that RAR-α is upregulated in several malignancies including Breast Cancer. It regulates granulocyte differentiation and has an association with the retinoic acid receptor signaling pathway and cellular response to estrogen stimulus. Furthermore, trifarotene was found as a potential synthetic compound that targets RAR-α through *in silico* and *in-vitro* study.

**Discussion:**

Overall, this research indicates that elevated expression of RAR-α enhances the onset of breast cancer. Using trifarotene medication to target RAR-α will significantly boost the response of breast cancer individuals to treatment and delay the development of resistance to drugs.

## Graphical Abstract

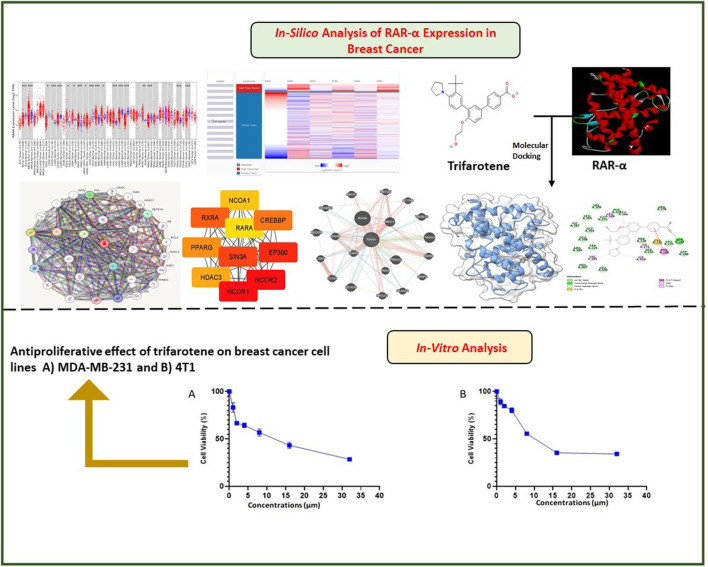

## Highlights

• This study reflects the pivotal role and prognostic consequence of RAR-α in BC pathogenesis.
• RAR-α was selected as a potent target for this study, as its overexpression accelerates Breast tumorigenesis.
• *In-vitro* and *in silico* outcomes reveal that RAR-α enhances tumorigenesis and exhibits worse clinical outcomes.
• Targeting RAR-α with therapeutic drugs will dramatically improve Breast cancer patients’ responses to therapy and prevent the emergence of chemoresistance.
• RAR-α acts as a prognostic marker and antagonizing it could be one of the potential therapeutic strategies to treat BC.

## 1 Introduction

The most frequent cancer diagnosed globally is now breast cancer (BC), surpassing lung carcinoma. In 2020, 2.3 million new instances of breast cancer were expected, accounting for roughly 11.7 percent of all new cancer cases, and 684,996 deaths were reported from it (Sung et al., 2021). Epidemiological research indicates that by 2030, there will be around two million people with breast cancer globally. In India, the incidence increased sharply, by about fifty percent, between 1965 and 1985 (Mehrotra and Yadav, 2022). There were an estimated 118000 incident cases in India in 2016, with 98.1 percent of the cases being women including the 526000 prevalent cases. From 1990 to 2016, there was a nationwide rise in the age-standardized incidence rate of BC in women over the preceding 26 years by 39.1 (Kaur et al., 2019). Global Cancer Observatory (GLOBOCAN) predictions for 2020 indicate that 19.3 million cancer cases occurred incidentally globally. India ranked 3^rd^ after China and the United States of America. By 2040, 2.08 million cancer cases are estimated to occur in India, indicating a 57.5 percent increase from 2020, according to GLOBOCAN’s prediction. Globocan data 2020 showed that breast carcinoma caused 10.6 percent (90408) of all deaths and 13.5 percent (178361) of all malignancy cases in India, respectively, with a 2.81 cumulative risk (Sofi et al., 2023). Although breast cancer is still the prominent reason for cancer death in females, recent data indicate a decrease in death rates (Printz, 2017). Increased knowledge, screening methods, and developments in targeted treatments are responsible for this. In contrast to morphological prognostic indicators, gene expression research has demonstrated that the tumor’s inherent molecular properties predominantly influence tumor response to therapy. As high-throughput platforms for gene expression research have emerged, molecular techniques like microarrays are utilized to investigate these inherent molecular characteristics (Weigelt et al., 2010). Therefore, a key element of treatment decision-making is the classification of BC into appropriate molecular subgroups. Perou and others categorised breast carcinoma into four major clinical subgroups based on receptor status ER, PR, HER2, gene expression patterns, and proliferative status as determined by Ki67 (Perou et al., 2000). These clinical subtypes include basal-like or basaloid or TNBC, HER2-enriched, and luminal (A and B). BCs with non-luminal A have a higher chance of recurring early. There is a prolonged dormancy period for potential recurrence in luminal-A tumors. TNBCs exhibit greater histological grade and advanced illness, making them aggressive (Jonnada et al., 2021).

Numerous risk factors, including genetic and inherited predisposition, are linked to the development of breast cancer. BCs are quite diverse. Treatment approaches for BC differ based on molecular characteristics, such as activation of the HER2, ER and PR hormonal receptors, gene mutations such as those in the BRCA1/2 and PIK3CA, and markers in the microenvironment of the immune system such as PDL1 and TIL. Early-stage BC is thought to be treatable and is treated mostly with local-regional treatments (surgery and radiation), with systemic therapy administered before or after surgery as needed (ACOG, 2019).

For the majority of early-stage HER2-positive and TNBC patients, preoperative or neoadjuvant therapy—such as immune checkpoint inhibitors or targeted therapy—is the accepted course of treatment. This is followed by risk-adapted post-surgical therapies. Endocrine treatment for five to 10 years is necessary for early breast cancer that is ER-positive. Presently, advanced BC with distant metastases is thought to be incurable. Systemic treatments in this case could involve immunotherapy, which is currently being used for TNBC, anti-HER2 targeted treatment for HER2 positive disease, poly (ADP-ribose) polymerase inhibitors for carriers of BRCA1/2 mutations, and endocrine therapy with targeted drugs like CDK4/6 inhibitors and PI3K inhibitors for hormone receptor + ve disease. In the future, precision medicine technologies may guide the escalation or de-escalation of personalized treatment (Hong and Xu, 2022). The development of therapeutic strategies for the treatment of BC and prognosis prediction depend heavily on the nuclear receptor superfamily of transcription factors, which include the ER and PR. There is mounting evidence that nuclear receptors other than ER and PR play a role in the initiation and progression of BC, according to recent research (Hua et al., 2009; Ni et al., 2011). Patients are frequently treated in clinics with medications that target nuclear receptors (Muscat et al., 2013). The anti-proliferative and anti-apoptotic effects of the glucocorticoid receptor in BC and its upregulation are linked to characteristics of extended survival (Abduljabbar et al., 2015).

Retinoids are both natural and synthetic compounds related to retinoic acids (RA), function through interacting with two different classes of nuclear receptors from the nuclear receptor superfamily: retinoic acid receptors (RAR-α, β, and γ) and retinoid X receptors (RXR-α, β, and γ) (Germain et al., 2006a; Pérez et al., 2012). These transcription factors are activated by ligands and belong to the superfamily of steroid hormone receptors. Retinoids are hypothesized to regulate cell development through both direct and indirect impacts on gene expression. Retinoic acid receptor (RAR-α, β, and γ), and retinoid X receptor (RXR-α, β, and γ) mediate these actions (Simeone and Tari, 2004). Retinoid receptors are essential for healthy development and are expressed by both healthy and malignant breast epithelial cells. Although the exact mechanism by which retinoid inhibits breast cell proliferation is yet unknown, it most likely involves several different signal transduction pathways. Several retinoids have the ability to bind to nuclear receptors and activate the AP-1 transcription pathway, which is triggered by growth factor signalling. Retinoids have a well-established function in the control of cell proliferation and differentiation, which is why numerous clinical trials are testing these drugs to prevent cancer (Decensi et al., 2003). Retinoids have been shown to prevent the development of cancer, slow the growth of tumors, and promote the differentiation and invasion of tumor cells in different tissues (Jan et al., 2023). Due to their capacity to prevent cell proliferation and induce morphological or phenotypic differentiation, retinoids, particularly RA, have been recommended as an adjuvant therapy for BC (Zanardi et al., 2006). A pleiotropic signaling protein called RA controls vital genetic programmes that regulate cell proliferation, differentiation, cell death, and/or survival (Clagett-Dame and Knutson, 2011; Samarut and Rochette-Egly, 2012). It also controls homeostasis, development, and other processes. Out of the three distinct RAR genes (RARα, RARβ, and RARγ) that have been identified, RARα interacts with target genes to contribute to tumor growth, metastasis, drug resistance, and other processes (Di Masi et al., 2015). According to the canonical model of RAR-mediated gene regulation, Retinoids (RXR) and RARα form heterodimers when ligand is absent. These heterodimers can engage constitutively specific response elements (RAREs) found in target gene promoters. RARα coupled to DNA is linked to the corepressors nuclear receptor corepressor 1 (NCoR1) and NCoR2, which serve as adaptors attracting additional subcomplexes with histone deacetylase (HDAC) activity, hence causing transcriptional repression (Perissi et al., 2010; Rochette-Egly, 2015). Following ligand binding, RARα experiences conformational changes that lead to its dissociation from corepressors and the recruitment of coactivators, including histone acetyltransferase (HAT) and nuclear receptor coactivator (NCoA)1/2/3. The recruitment of transcriptional machinery and the activation of target genes are made possible by this mechanism (Glass and Rosenfeld, 2000).They control the expression of certain target gene subsets related to apoptosis, proliferation, and cellular differentiation (Bour et al., 2006; Eifert et al., 2006; Delacroix et al., 2010; Gudas and Wagner, 2011). Accordingly, RARs are essential for many biological processes, such as development, immunity, reproduction, organogenesis, and homeostasis. This is demonstrated by pharmacological, vitamin A deficiency, and genetic research carried out in mice (Niederreither and Dollé, 2008; Su and Gudas, 2008; Dollé, 2009; Mark et al., 2009). Apart from their physiological functions, RARs are also linked to pathologies and tumors as a result of mutations, protein fusions, changes in expression levels, or abnormal post-translational changes that lead to aberrant functions and homeostasis breakdown. Given their capacity to control development and differentiation throughout life, it is obvious that aberrant expression of RAR and/or function may play a role in many malignancies. Tumor growth has thus been linked to RAR overexpression, deletion, mutations, or abnormal post-transcriptional alterations (Duong and Rochette-Egly, 2011). The retinoic acid receptor RAR-α is over-expressed in several malignancies. Increased invasion and migration in multiple cancer forms, such as HNSC carcinoma, pediatric low-grade gliomas, lung adenocarcinoma, and breast cancer, have been associated with its overexpression. Numerous authorised therapeutic regimens targeting RAR-α have been developed, improving patient survival rates (Jan et al., 2023). Identification of patient subpopulations who may be responsive to therapy and hence would benefit from it is necessary for the successful therapeutic usage of retinoids in the treatment of BC. High RAR-α levels in the tumor are associated with sensitivity to retinoid therapy, according to preclinical and clinical findings (Centritto et al., 2015). Co-amplification of the RAR-α gene, which results in enhanced production of the RAR-α protein is shown in a significant fraction of HER2 positive BCs. RA can effectively inhibit cell proliferation in these cases. In the context of ER negative cancers, which do not respond to hormonal treatments, this is especially important. Anti-HER2 positive medications are more effective in ER-negative/HER2positive/RAR-α+ tumors when RA is given at the same time (Paroni et al., 2012).

Trifarotene, a 4^th^ generation retinoid, on a topical basis, is used to treat acne vulgaris clinically. Compared to other retinoids trifarotene has a low toxicity and good stability, according to a recent study that examined its metabolic and pharmacological characteristics (Naik, 2022). Trifarotene has anti-inflammatory characteristics, is highly selective for RARγ, and regulates processes involved in the etiology of skin cancer (such as differentiation, proliferation, etc.). Additionally, this medication is well tolerated and relatively easily metabolized in the liver (Aubert et al., 2018; Ramchatesingh et al., 2022). Accordingly, trifarotene in the context of drug repurposing can be used in treating breast carcinoma including other cancers.

In the current work, for *in silico* investigation we explored the RAR-α expression and prognostic significance in breast carcinoma using bioinformatics tools. The expression pattern, genetic alteration, prognostic relevance, and immunological association of RAR-α in different BC tissues were assessed using the TIMER 2.0, UCSC XENA, Bc GeneExminer, ENRICHR, TISCH, UALCAN, KM-Plotter and Oncomine tools. In breast cancer tissues, RAR-α levels were elevated, and overexpression of RAR-α was linked with worse overall survival (OS) and relapse-free survival (RFS). Additionally, in-silico docking of RAR-α with trifarotene was conducted and showed a high binding score. Furthermore, trifarotene's effect was subsequently investigated *in-vitro* in breast cancer cells. This study shows that RAR-α is essential for breast tumorigenicity since it is linked with worse OS and RFS in breast carcinoma individuals with high RAR-α expression. Therefore, to reduce the chemoresistance development and improve the response of BC individuals to treatment can be achieved by targeting RAR-α in conjunction with other cancer pathways.

## 2 Materials and methods

### 2.1 Expression pattern of RAR-α in PAN cancer

The comprehensive online portal TIMER 2.0 (http://timer.cistrome.org/) is utilised to investigate immune infiltrates and expression patterns in a variety of cancer types. Using TCGA datasets, this web portal was utilised to investigate the expression pattern of RAR-α expression in different types of cancers.

### 2.2 UCSC XENA

A heatmap was generated by the examination of the expression of several RARs in BC using the UCSC XENA online web source. Users can investigate functional genomic data sets and search for relationships between phenotypic and/or genomic characteristics using UCSC Xena (Goldman et al., 2020).

### 2.3 UALCAN

To assess the RAR-α expression in various subclasses, age ranges, and ethnicities of breast cancer individuals, we utilised the database UALCAN, a comprehensive online tool used to assess cancer OMICS data (Chandrashekar et al., 2017). Further to explore the somatic alteration at pathway-level in RAR-α across tumors comprising key pathways for different types of malignancies, UALCAN along with whole-exome, CNA data, and coupled proteomic was employed.

#### 2.3.1 bc-GenEXMiner

A web-based bioinformatics tool, namely, bc-GenEXMiner v5.0, was employed for annotated breast cancer transcriptome data to investigate the association between RAR-α expression and other clinical characteristics of breast cancer individuals, such as the status of hormone receptors, nodal status, NPI status, and SBR grade (Jézéquel et al., 2012).

### 2.4 GeneMANIA

GeneMANIA (https://genemania.org/) determines new genes linked to a set of input genes by using a vast library of functional association data (Warde-Farley et al., 2010). Co-expression, co-localization, pathways, genetic and protein domain similarities, and co-expression are a few types of association data. To predict RAR-α′s function and association in the gene-gene interaction (GGI) investigation, we employed the web resource GeneMANIA. This free resource offers a versatile and easy-to-use interface for determining the function of gene sets and genes of interest. The function of RAR-α and its correlation with other important genes were predicted using the database.

### 2.5 Protein-protein interaction (PPI) analysis

A 0.7 confidence level PPI network of RAR-α was constructed using the internet database STRING (http://stringdb.org). An online biological resource called STRING was developed to create and research functional connections between proteins (Szklarczyk et al., 2015). Using the Cytoscape programme, the PPI web was visualised and examined in more detail (version) (Smoot et al., 2011). The significant modules of the PPI network were explored with the aid of the MCODE (Molecular Complex Detection) plug-in for the Cytoscape software. The PPI web’s top ten HUB nodes were examined using the Cytohubba plugin option (Chin et al., 2014).

### 2.6 RAR-α analysis in single-cell sequencing database (TME)

In order to observe significantly aberrant RAR-α expression in various datasets in breast cancer tissues, TISCH was utilised (Sun et al., 2021). We examined the expression of RAR-α in primary and metastatic BC datasets to ascertain the relationship between RAR-α expression patterns and tumor growth.

### 2.7 RAR-α gene ontology (GO) and its pathway enrichment

GO is one of the important bioinformatics databases. The main objective of this database is linking the gene and gene product features in all species. It gives us a gradually compiled list of thousands of organised terms for molecular processes, cellular parts, and biological activities. Additionally, it curates and forecasts gene annotations with a *p*-value of 0.05. Enricher (http://amp.pharm.mssm.edu/Enrichr), a comprehensive online resource that gathers biological information for future biological findings, examined the GO annotation investigation. Further KEGG pathway analysis was also done utilising Enricher (Kanehisa et al., 2017).

### 2.8 Docking of trifarotene and RAR-α

#### 2.8.1 Selection of target and its preparation

Using ID 5K13, the target protein RAR-α was acquired in PDB format from the Protein Data Bank. In discovery studio the function ‘Prepare protein’ was used to standardize the name of atom, elimination of disordered alternate conformations, addition of missing main- and sidechain atoms, and simplify the structure for RAR-α. Moreover, heteroatoms and H_2_O molecules were eliminated.

#### 2.8.2 Molecular docking

This study aims to provide insights into trifarotene’s affinity for RAR-α binding. The molecules in question were subjected to docking investigations using Auto dock v 4.2.6. To compute the binding cavity of proteins, the pre-calculated co-crystallized X-ray structure from the RCSB PDB was utilized. To calculate the residue positions within a 3Å radius, the co-crystallized ligand was utilized. Chimera (https://www.cgl.ucsf.edu/chimera/) was utilized in the cavity selection process to eliminate co-crystallized ligands, and the steepest descent and conjugate gradient methods were then applied to decrease energy. Both the receptor and the target molecule were saved in pdbqt format after combining non-polar hydrogens. For molecular docking, a 49.4 × 49.9 × 56.9 Å grid box was utilised. Grid boxes had to be designed with specific measurements and a 0.3 Å spacing. The Lamarckian Genetic Algorithm was used in protein-ligand complex docking investigations. Molecular docking investigations were conducted in triplicate, with each replicate having 50 solutions, a population size of 500, 2500000 assessments, a maximum generational number of 27, and all other parameters set to their default levels. The best cluster with the lowest energy score and the greatest number of populations was identified by re-clustering with the clustering tolerances of 0.25, 0.50, and 1 after the docking process was finished. This produced the RMSD clustering maps.

#### 2.8.3 Molecular dynamics simulation

The Desmond 2020.1 was used to conduct MD simulations (Shaw et al., 2010), on 5K13M + trifarotene complex. Within this system, the OPLS-2005 force field (Bowers et al., 2006; Chow et al., 2008; Shivakumar et al., 2010) and explicit solvent model containing the SPC H_2_O molecules (Jorgensen et al., 1983) were used in a timeframe salvation box with dimensions of 1.0 Å × 1.0 Å x 1.0 Å. Sodium ions (Na+) were added to neutralize the electrical charge. The apparatus was filled with a solution of 0.15 M sodium chloride in order to replicate the metabolic environment. The protein-ligand complexes were first exposed to an NVT ensemble for 10 nanoseconds in order to retrain the system. After the previous process, a 12-ns equilibration and minimization run were performed using an NPT ensemble. The Nose-Hoover chain coupling method was used to create the NPT ensemble (Martyna et al., 1994). Every model had its temperature adjusted while keeping the pressure at 1 bar and the relaxation time at 1.0 picoseconds constant. In the simulation, a temporal increment of 2 femtoseconds was used. The pressure was controlled using the barostat technique (Martyna et al., 1992) of the Martyna-Tuckerman-Klein chain coupling scheme. Two picoseconds were selected as the relaxation time. To calculate long-range electrostatic interactions, we used the Ewald particle mesh method (Toukmaji and Board, 1996). For the coulomb interactions, the radius was kept at 9Å constant. The RESPA integrator was utilised to calculate the bonded forces for every trajectory, with a time step of 2 fs. One hundred nanoseconds was the temporal duration per unit for the most recent manufacturing run. Several important metrics, such as the RMSD, Rg, RMSF, and number of hydrogen bonds, were measured in order to evaluate the stability of the molecular dynamic’s simulations. The aforementioned values were utilised to evaluate the molecular dynamics simulations’ stability.

### 2.9 MTT assay

Breast cancer B cells (MDA-MB-231 and 4T1) were seeded in a 96-well plate at the seeding density of 3 × 10^3^ cells per well. After 24 h, different dosages of trifarotene were given to cells, and in a humidified CO2 incubator they were cultured for 72 h. After 72 h, the Invitrogen MTT assay kit was used to perform the assay. For the assay, the manufacturer’s protocol was followed appropriately (Van Meerloo et al., 2011).

### 2.10 Statistical significance

The statistical analysis was done using one-way or two-way ANOVA in GraphPad Prism V 8.43, followed by Tukey multiple comparisons test. *p* < 0.05 was considered significant.

## 3 Results

### 3.1 RAR-α is overexpressed in various cancers

**FIGURE 1:**
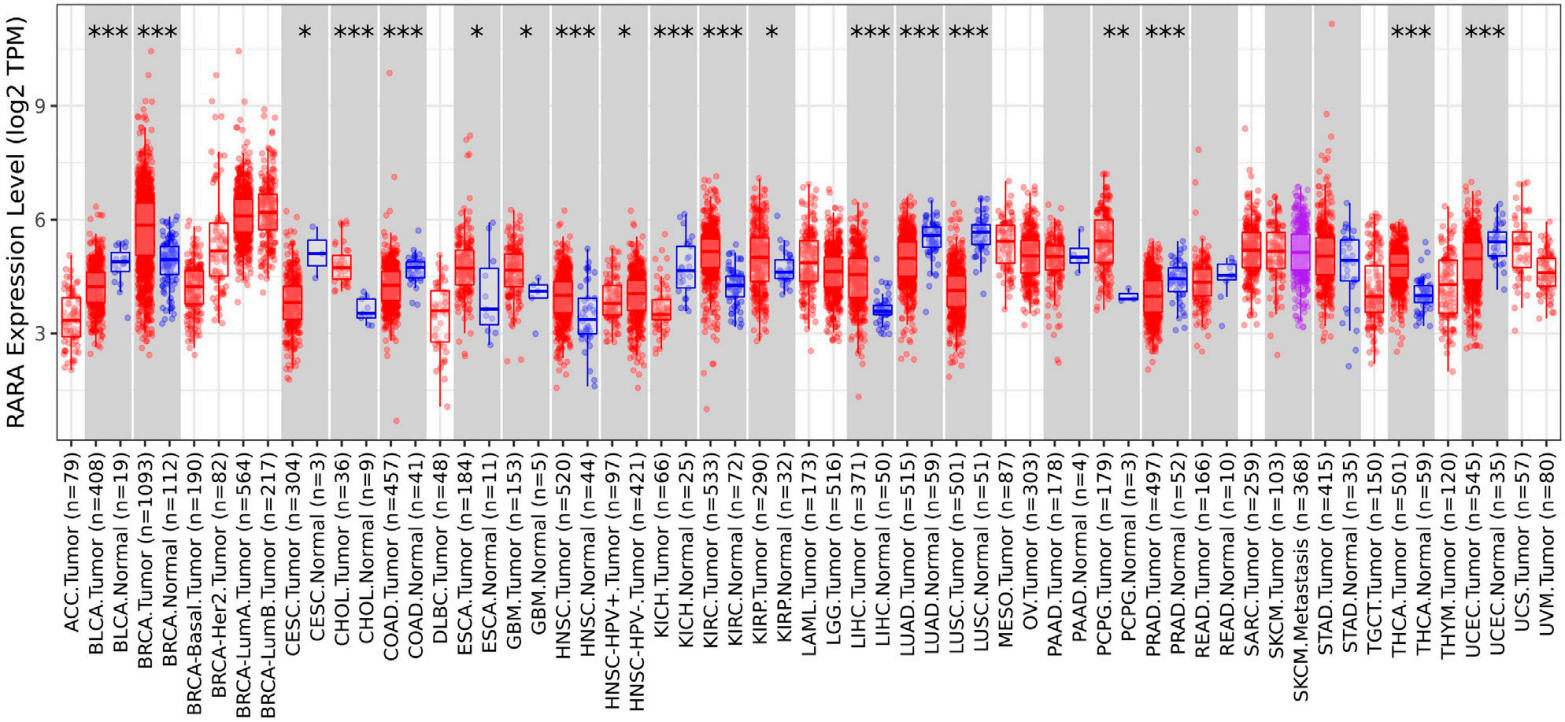
RAR-α expression in many cancers using the box plot model from the TIMER Database. When compared to other tumors, RAR-α expression is highest in BC, whereas it is considerably enhanced in the majority of malignancies including thyroid cancer (THCA), sarcoma (SARC), lung adenocarcinoma (LUAD), head and neck squamous cell carcinoma (HNSC), lung squamous cell carcinoma (LUSC), kidney renal clear cell carcinoma (KIRC), uterine corpus endometrial carcinoma (UCEC).

The TIMER 2.0 database was utilised to ascertain the expression level of RAR-α in several types of malignancies. TIMER 2.0 study demonstrates the high expression of RAR-α in several malignancies. According to the box plots RAR-α is elevated in many malignancies, including BC, LGG, THCA, SARC, LUAD, HNSC, LUSC, KIRC, and UCEC (Figure 1). Patients with breast cancer showed the highest expression of RAR-α among these malignancies, followed by those with Luminal-A BC.

### 3.2 RAR-α unlike other RARs is overexpressed in BC

**FIGURE 2:**
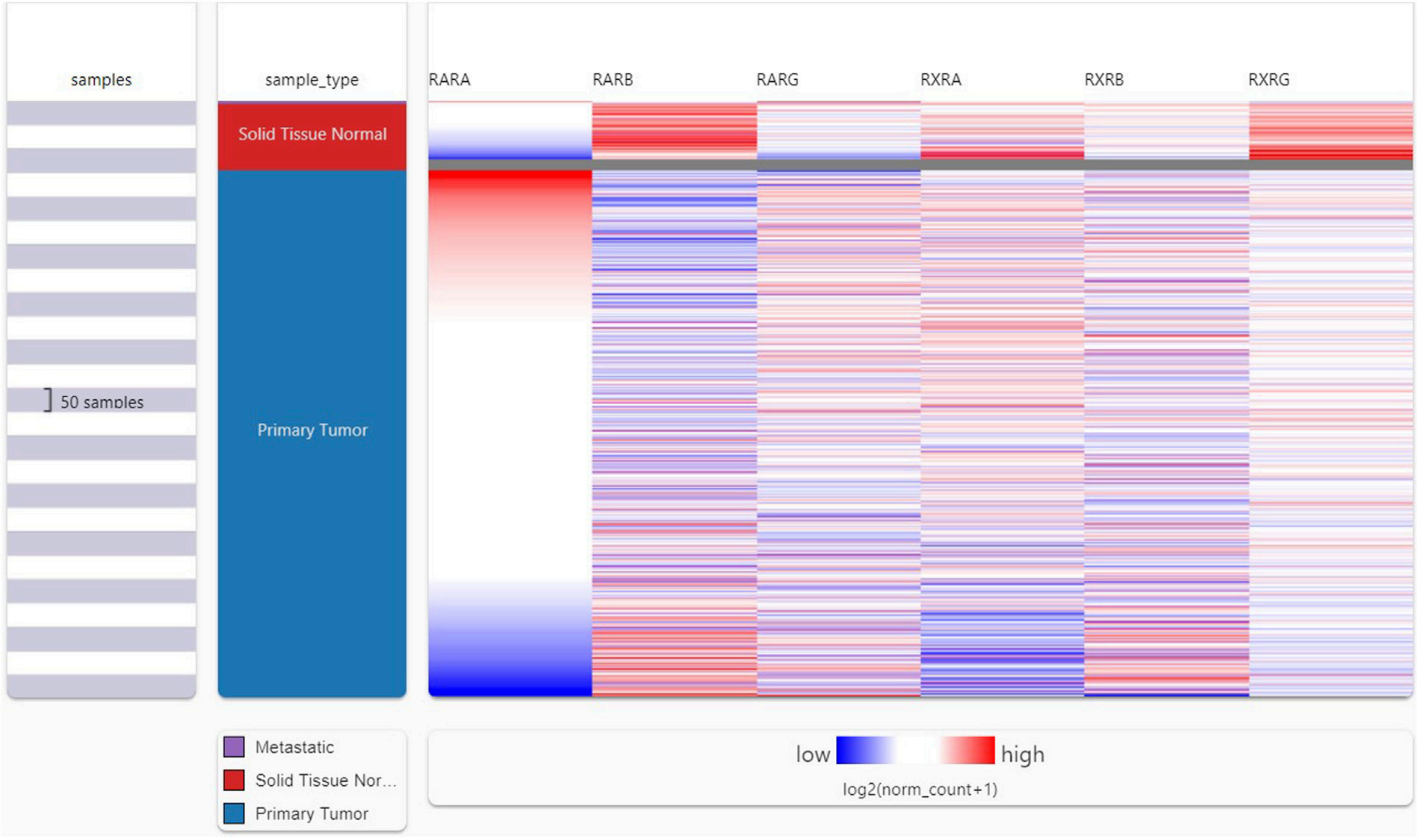
UCSC XENA heat map showing several RARs and RXRs in BC. RAR-α was considerably elevated in BC patients in contrast to other RARs (RAR-β, RAR-γ) and RXRs (RXR-α, RXR-β, RXR-γ).

Following the analysis of the RAR-α expression profile in many malignancies, we proceeded to create a heat map featuring crucial RARs and RXRs that are vital in the development of BC. A heat map of important RARs and RXRs in breast cancer was created from UCSC Xena (Figure 2). The heat map’s findings also demonstrated that, in comparison to other RARs and RXRs, RAR-α is significantly overexpressed in breast cancer.

### 3.3 RAR-α is highly overexpressed in BC patients

**FIGURE 3:**
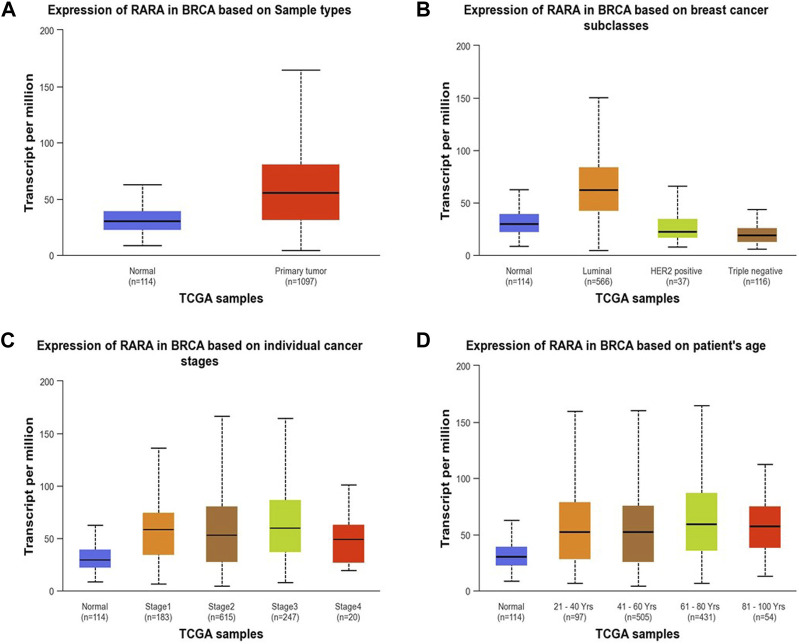
RAR-α expression profile of patients with BC. In patients with primary tumors, the expression of RAR-α was elevated **(A)** in women of 61–80 years of age **(D)**. Patients with Luminal BC exhibited high expression **(B)** and in stage 3 breast cancer the expression of RAR-α was elevated **(C)**.

RAR-α expression analysis in relation to type of sample, age group, subtypes of breast cancer, and ethnicity was further examined using the UALCAN database (Figure 3). The results of the investigation showed that primary tumors had higher levels of RAR-α expression than normal patients (Figure 3A). The patients with luminal breast cancer displayed increased expression of RAR-α when compared to HER2 or TNBC subtypes (Figure 3B). According to the cancer stage, stage 3 showed the highest expression of RAR-α, followed by stage 2, stage 1, and stage 4 (Figure 3C). Additionally, RAR-α expression were augmented in the age group of sixty-one to Eighty years old women (Figure 3D).

**FIGURE 4:**
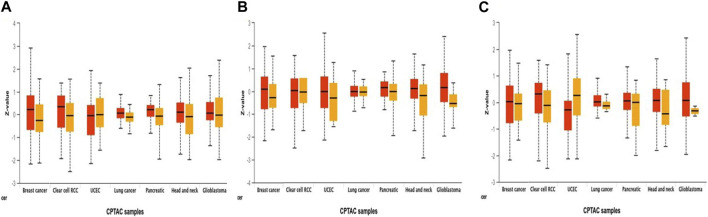
Pathway status association with the expression of RAR-α in pan-cancer. Through the UALCAN portal, RAR-α expression was examined for modulation in signaling pathways through, **(A)** Wnt signaling, **(B)** mTOR pathway, and **(C)** p53/Rb-related pathway (*p*-value < 0.01).

Additionally, using combined proteomic, whole-exome, and CNA data, we thoroughly investigated somatic changes at pathway-level in RAR-α across tumors, identifying significant pathways across various types of malignancies. It was found that BC patients with overexpression of RAR-α had aberrant signaling pathways including WNT, mTOR, and the p53-Rb (Figure 4).

### 3.4 Overexpression of RAR-α positively correlates with ER + ve and PR + ve status

**FIGURE 5:**
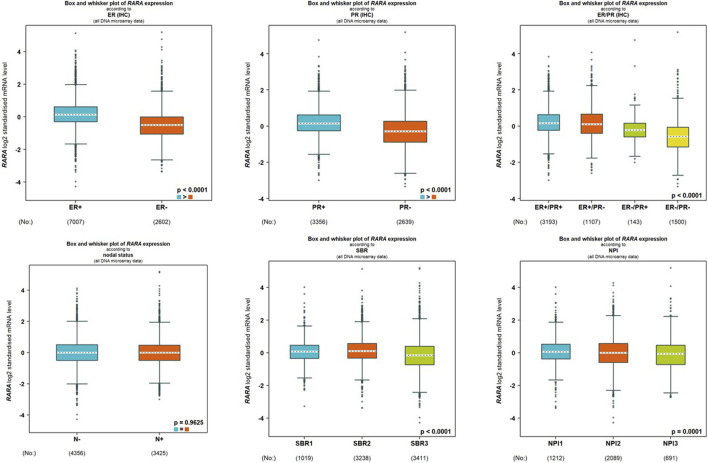
RAR-α expression is associated with several clinicopathological features. The high expression of RAR-α is linked with ER positive, PR positive, ER positive/PR positive, Nodal negative, SBR2 and NPI2 status.

We analyzed the connection between RAR-α and clinicopathological features of BC people using the bc-GenEXMiner portal. In breast cancer individuals with +ve status of oestrogen and progesterone and ER positive/PR positive receptor expression, RAR-α is usually expressed at higher levels. Furthermore, compared to nodal positive patients, those with nodal negative status exhibit higher levels of RAR-α expression. Also, the SBR2 and NPI2 grades showed significant expression of RAR-α (Figure 5).

### 3.5 RAR-α and RXR-α have a strong correlation

**FIGURE 6:**
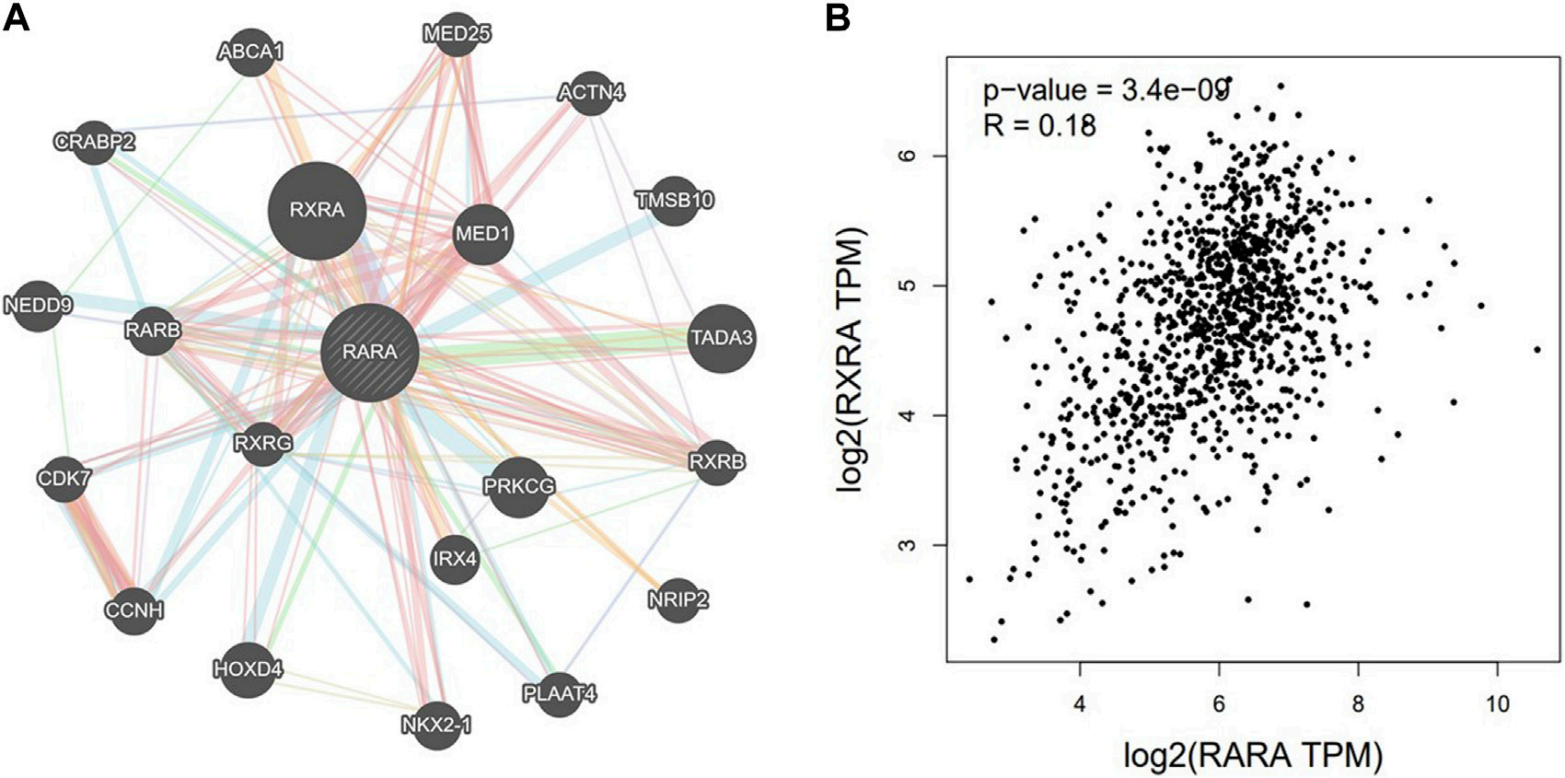
**(A)** GGI network of RAR-α using GeneMANIA, RAR-α depicted a high correlation with RXR-α followed by TADA3, MED1, HOXD4, TMSBI10, and several other genes. **(B)** With an R-value of 0.18, the Gepia2 database showed a strong association between RAR-α and RXR-α in BC.

Using the GeneMANIA portal, we discovered the network of gene-gene interactions of RAR-α (Figure 6). As seen in, RAR-α showed strong interactions with RXR-α, which was followed by TADA3, MED1, HOXD4, TMSBI10, and several additional genes (Figure 6A). The GGI network’s interactions were based on physical connections, co-localization, co-expression, and pathways of genetic interaction. Gepia2 was used to analyse the connection between RAR-α and RXR-α. The results showed that RAR-α and RXR-α are strongly correlated in individuals with breast cancer having an R-value of 0.18. (Figure 6B).

### 3.6 RAR-α protein-protein interaction (PPI)

**FIGURE 7:**
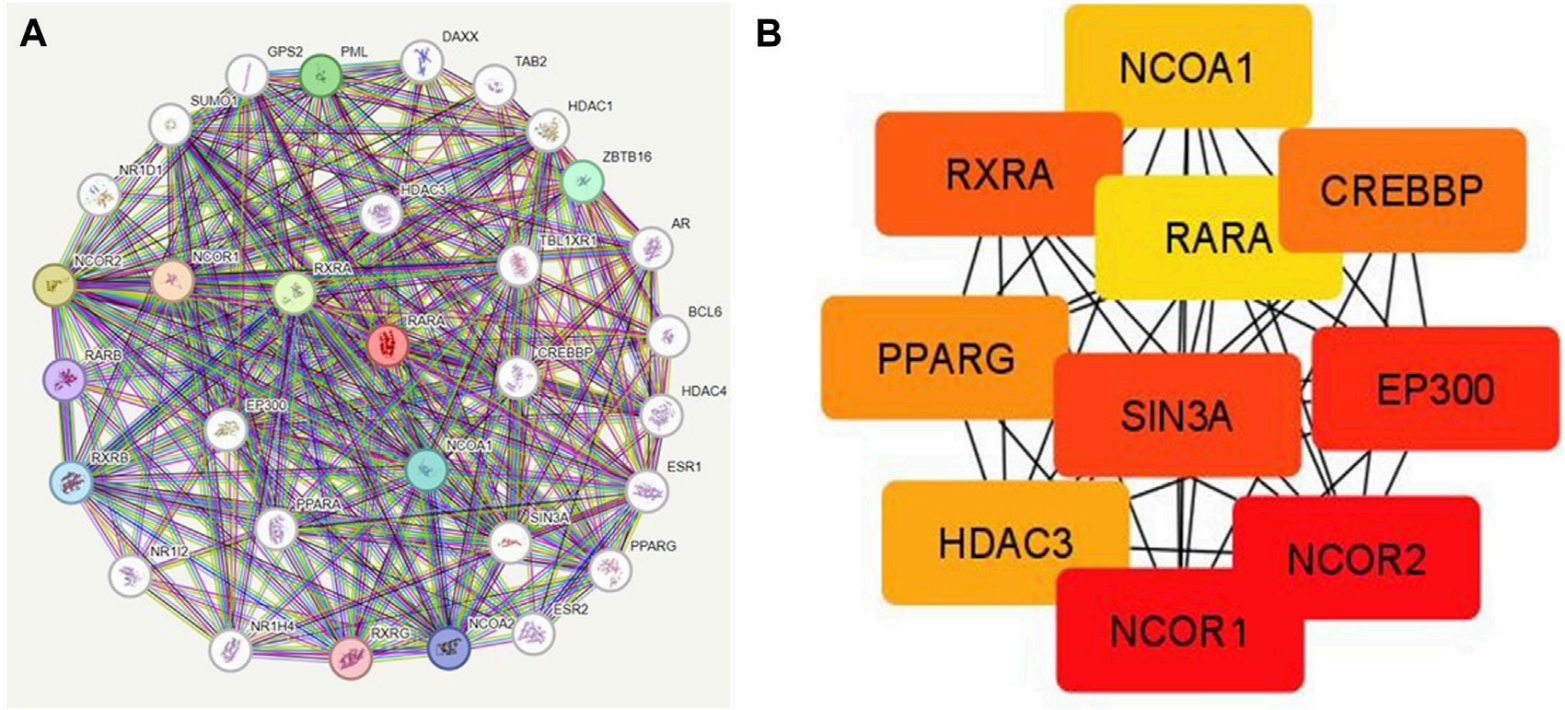
**(A)** PPI interactions of RAR-α with other proteins using string database. **(B)** With Cytohubba, the network’s top 10 hub genes based on their degree score were determined. The ten genes of the network included RARA, RXRA, NCOA1, NCOR1, NCOR2, PPARG, HDAC3, CREBBP, EP300, and SIN3A.

PPI was created by connecting co-expressed thirty-one genes (nodes) with 282 proteins via the STRING portal (edges) (Figure 7). Also, the PPI network showed an average local clustering coefficient: 0.794, average node degree: 18.2, PPI enrichment *p*-value: <1.0e-16, and an expected number of edges: 70 (Figure 7A). Cytohubba was used to identify the top ten hub genes on the web based on degree score, as demonstrated in (Figure 7B). The top ten genes of the network comprised RARA, RXRA, NCOA1, NCOR1, NCOR2, PPARG, HDAC3, CREBBP, EP300, and SIN3A (Figure 7B). The MCODE plug-in for Cytoscape programme was analysed in order to extract the most significant component of the PPI network.

### 3.7 Association of RAR-α with tumor stroma

**FIGURE 8:**
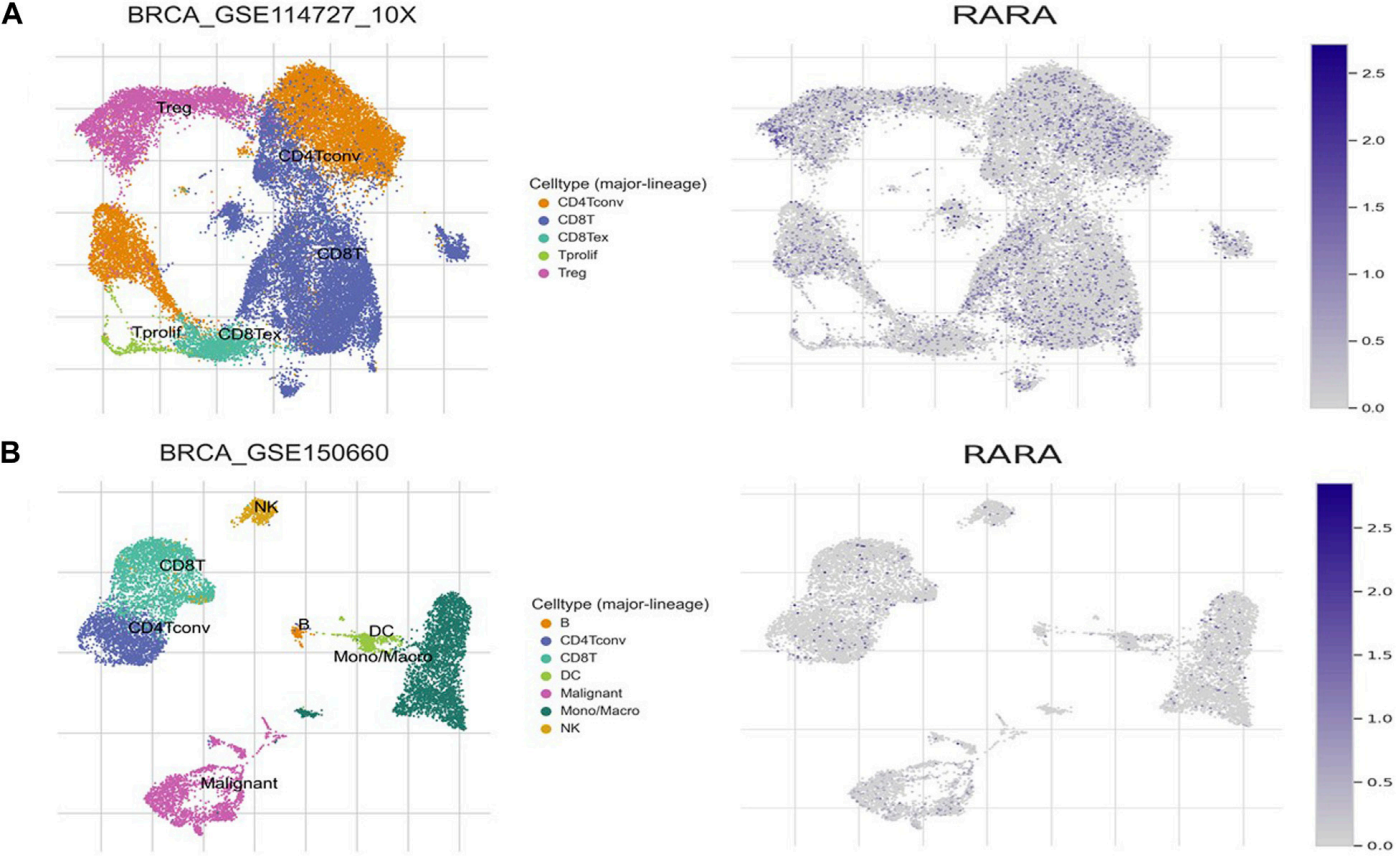
Expression pattern of RAR-α in BC using single-cell RNA-seq database TISCH. **(A)** Primary tumor of the breast, **(B)** Metastatic BC. The expression of RAR-α was highly enriched in Treg cells, CD4, CD8, Tprolif cells and malignant cells.

We analysed the aberrant expression profile of RAR-α in database TISCH, which focuses on tumor microenvironment. Several BC datasets containing data on primary and metastatic cancers were subjected to expression pattern analysis. The study revealed that RAR-α is greatly elevated in primary cancers than metastatic cancers. Furthermore, the primary tumor cell population’s expression patterns for RAR-α expression varied greatly. CD4T and CD8T cells expressed higher RAR-α expression in BC patients with primary tumors (Figure 8).

### 3.8 Gene ontology and KEGG analysis

**FIGURE 9:**
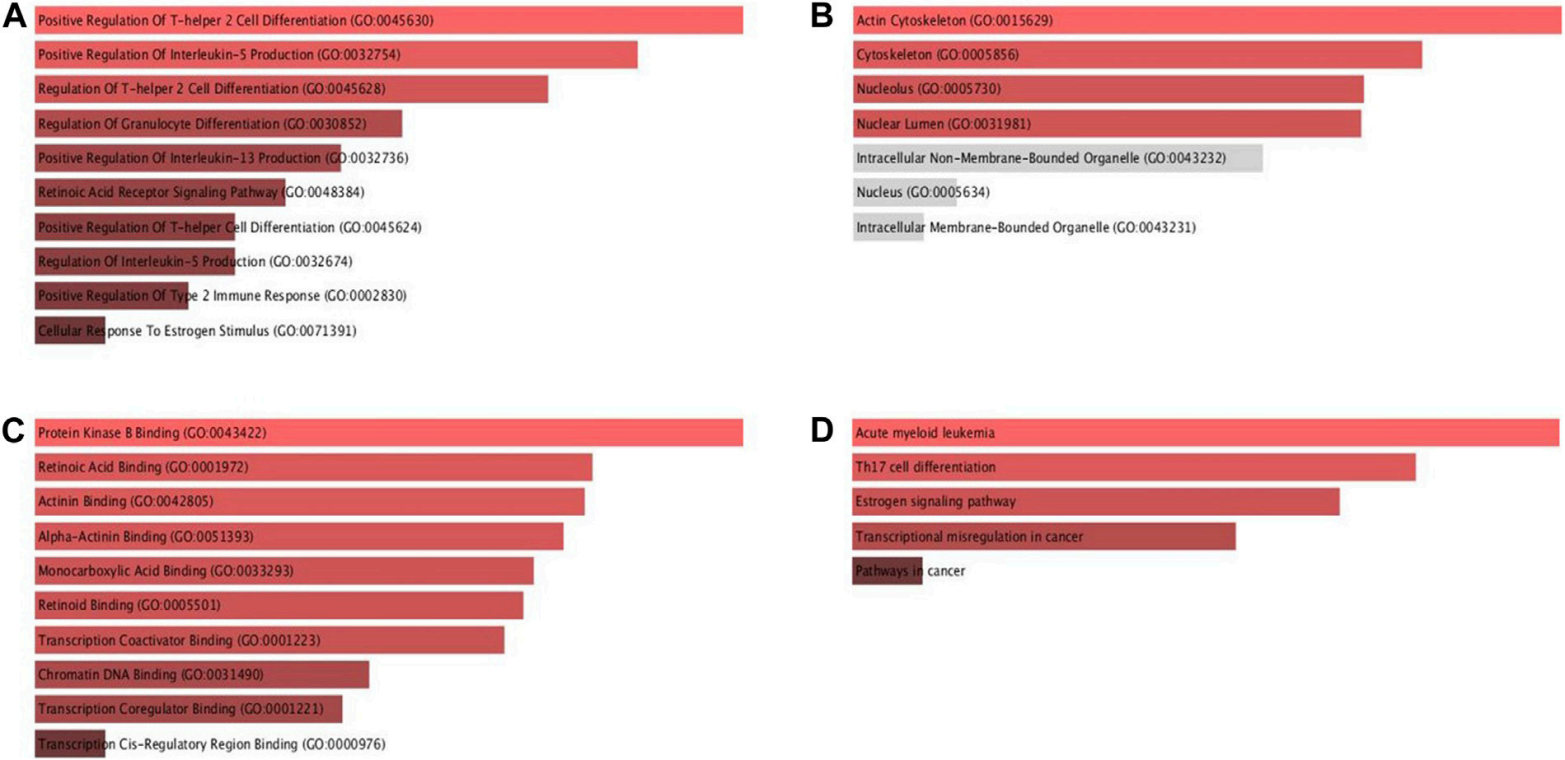
GO and KEGG pathway analysis of RAR-α using the ENRICHR database. **(A)** Among the biological processes, RAR-α was found to be associated with the positive regulation of T-helper two cell differentiation, IL-13 production, IL-5 production including others. **(B)** In cellular compartment RAR-α was more abundant in the actin cytoskeleton followed by the cytoskeleton, nucleolus, and nuclear lumen. **(C)** Among molecular functions, RAR-α was involved in retinoic acid binding, actinin binding, transcription coactivator binding, chromatin DNA binding, including others. **(D)** The KEGG analysis showed that RAR-α is linked with acute myeloid leukemia, Th17 cell differentiation, estrogen signaling pathway, transcriptional mis-regulation in cancer including pathways in cancer.

The Enrichr database was utilised for GO and KEGG analysis (Figure 9). Among the biological processes, RAR-α was found to be associated with the positive regulation of T-helper two cell differentiation, IL-13 production, IL-5 production, T-helper cell differentiation, and type 2 immune response. In addition, RAR-α also regulates granulocyte differentiation and has an association with the retinoic acid receptor signaling pathway and cellular response to estrogen stimulus (Figure 9A). RAR-α was more abundant in the actin cytoskeleton followed by the cytoskeleton, nucleolus, and nuclear lumen among the cellular compartment (Figure 9B). Among molecular functions, RAR-α was involved in protein kinase B binding, retinoic acid binding, actinin binding, alpha-actinin binding, monocarboxylic acid binding, retinoid binding, transcription coactivator binding, chromatin DNA binding and transcription coregulator binding (Figure 9C). The KEGG pathway study revealed that RAR-α is related with acute myeloid leukemia, Th17 cell differentiation, estrogen signaling pathway, transcriptional mis regulation in cancer including pathways in cancer (Figure 9D).

### 3.9 Molecular docking showed high binding of trifarotene with RAR-α

#### 3.9.1 Molecular docking

**FIGURE 10:**
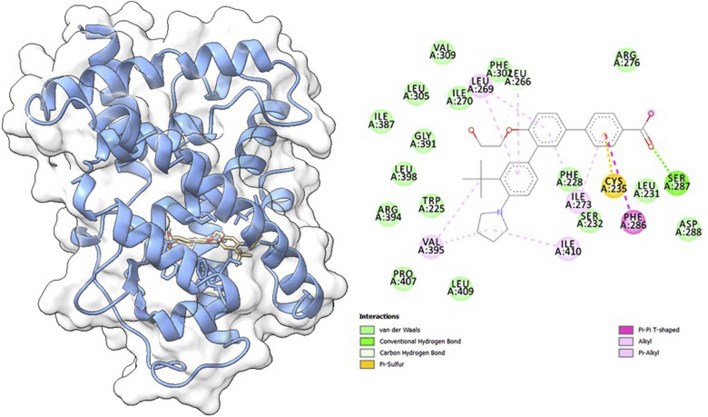
The molecular surface view of the RAR-α with trifarotene bonded in a deep cavity. Two-dimensional interaction displays the interactions between a protein and ligand, represented by dotted lines.

Molecular docking studies were carried out to understand the binding properties of 5K13M complex with trifarotene. Figure 10 displays docked complexes, molecular surfaces, and 2D and 3D interactive graphs. With the lowest binding energy between 5K13M with trifarotene showed a remarkable binding affinity ΔG (−8.35) kcal/mol. During the interaction of the trifarotene with 5K13M exhibited conventional hydrogen bond with Ser287, pi-pi T shaped interaction with Phe286, pi-sulfur with Cys235, pi and Pi-alkyl interactions are observed with Leu266, Leu269 and Ile270 residues (Figure 10).

#### 3.9.2 Molecular dynamics simulation (MDS)

**FIGURE 11:**
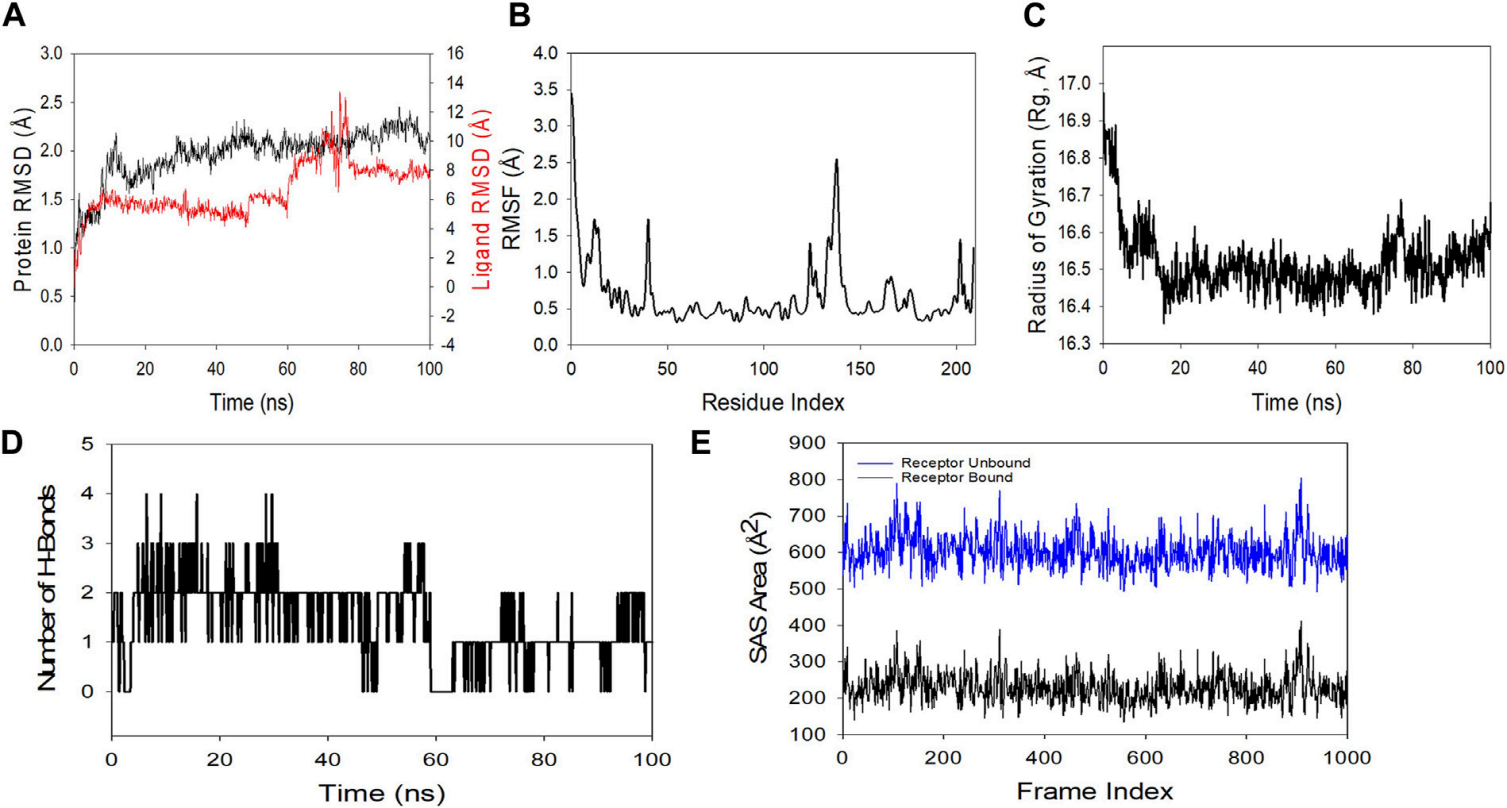
Using MD simulation method, analyzing the 100 ns trajectories of **(A)** the Cα backbone RMSD of 5K13M + trifarotene, **(B)** RMSF of the Cα backbone of 5K13M + Trifarotene, **(C)** Cα backbone Rg of 5K13M + trifarotene, and **(D)** hydrogen bond formation in 5K13M + trifarotene **(E)** Surface area accessible to solvents (5K13M + trifarotene).

MDS studies were performed to determine 5K13M + trifarotene’s stability and convergence. Comparing the RMSD, the 100 ns simulation showed nearly stable conformation (RMSD). The Cα-backbone RMSD of 5K13M + trifarotene showed a more stable 2.0 Å. The Trifarotene exhibited large conformational changes and thus the RMSD till 50 ns was constant then overshoot and aligned at 8 Å (Figure 11A). A consistent RMSD plot during simulation indicates stable protein-ligand conformations and satisfactory convergence. Consequently, it can be hypothesised that the protein’s greater affinity for trifarotene makes it quite stable in complex. Large fluctuations in protein bound to trifarotene were seen in the RMSF plot at residues 5–20, 48, 115–125, and 130–148. These fluctuations may have been caused by the residues’ increased flexibility (Figure 11B). Over the course of the 100 ns simulation, the majority of residues showed less fluctuation, suggesting that the amino acid conformations were rigid. The protein’s compactness is measured by its Rg. The 5O79 Cα-backbone linked to trifarotene in this investigation showed a decrease in Rg, from 16.9 to 16.54 Å (Figure 11C). A decrease in Rg signifies a highly compact orientation of the protein when it is coupled to a ligand. The complexity and stability of the complex are indicated by the number of H bonds between the protein and ligand. The number of H bonds between 5O79 and trifarotene showed couple of hydrogen bonds observed on an average upto 100 ns (Figure 11D).

Similar results were also seen in SASA in both the ligand-bound and unbound states, as confirmed by Rg analysis. (Figure 11E) makes it evident that in the unbound state of trifarotene to receptor protein 5O79, high surface area accessible to solvent was present throughout every case (Figure 11E). When bound with ligands, the SASA value decreased in comparison to the unbound condition (Figure 11E). More lowering of peak is more tightly bound conformation. The general analysis of Rg suggests that the binding of the ligands induces the corresponding proteins to compress.

#### 3.9.3 Molecular mechanics generalized born surface area (MM-GBSA) calculations

MDS trajectory was utilised to get the binding free energy and additional contributing energy in the form of MM-GBSA for the complex of 5O79 and trifarotene. The findings (Table 1) indicated that ΔGbindCoulomb, ΔGbindvdW, and ΔGbindLipo contributed the most to ΔGbind in the stability of the simulated complexes, but ΔGbindCovalent and ΔGbindSolvGB contributed to the instability of the corresponding complexes. 5K13M + trifarotene complex have remarkably higher binding free energies (Table 1). In addition to having a strong affinity for proteins, trifarotene also binds to the chosen proteins effectively and can create stable protein-ligand complexes.

**TABLE 1:** Binding free energy components for the 5K13M + Trifarotene calculated from MM-GBSA.

*Energies (kcal/mol)*	*5K13M + Trifarotene*
*ΔG*_*bind*_	−77.35
*ΔG*_*bind*_ *Lipo*	−10.75
*ΔG*_*bind*_ *vdW*	−45.60
*ΔG*_*bind*_ *Coulomb*	−39.68
*ΔG*_*bind*_ *H*_*bond*_	−5.14
*ΔG*_*bind*_ *SolvGB*	25.56
*ΔG*_*bind*_ *Covalent*	1.72

### 3.10 Trifarotene possesses potent anticancer activity

**FIGURE 12:**
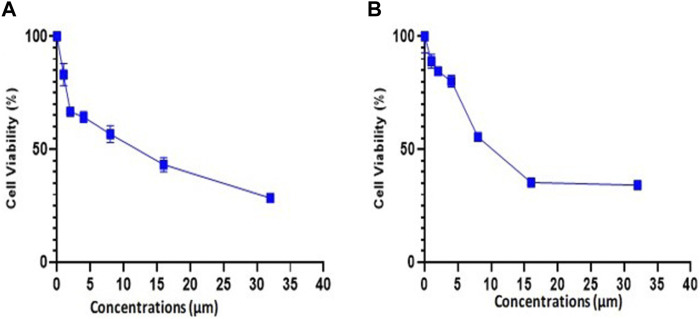
Trifarotene’s anti-proliferative activity on breast cancer cell lines; **(A)** MDA-MB 231 cells, **(B)** 4T1 Cells. The cells were treated with different concentrations of trifarotene. The cell viability was determined by MTT test after 72 h.

The experiment for cell viability was performed on MDA-MB-231 and 4T1 cells to assess the anticancer activity and verify the results of docking. BC cell lines (MDA-MB-231 and 4T1 cells) were seeded in a 96-well plate and were treated with trifarotene in a dose-dependent manner. Trifarotene showed significant anticancer activity with an IC50 Value of 9.558 µM and 11.63 µM in cell lines MDA-MB-231 and 4T1 respectively. The low IC50 value indicates that trifarotene significantly inhibits the proliferation of cancer cell (Figure 12). Furthermore, compared to 4T1 cells, MDA-MB-231 cells showed a remarkable sensitivity to trifarotene.

## 4 Discussion

Cancer is the 2^nd^ main reason for mortality and a significant public health issue (Sofi et al., 2022). Breast cancer (BC), particularly has a significant occurrence and mortality rate, making it the most widespread form of malignancy among females globally. It is a type of cancer connected to a substantial number of deaths. The primary reason behind this elevated mortality is the emergence of drug resistance, which poses a major obstacle in the treatment of BC (Correia et al., 2018). The OS and RFS of BC patients have improved due to advancements in early detection and therapy, but the rise in metastatic and resistant malignancies need immediate attention. Thus, there is an urgent need to investigate new therapeutic targets and drug repurposing for established breast cancer targets (dos Santos Correia, 2019). RARs form heterodimers with RXRs, which are ligand-dependent transcriptional regulators (Germain et al., 2006b; Germain et al., 2006c). RARs regulates numerous biological functions, including development, reproduction, immunity, organogenesis and homeostasis. Apart from their physiological functions, RARs are also linked to pathologies and tumors as a result of mutations, protein fusions, changes in expression levels, or abnormal post-translational changes that lead to altered functions and homeostasis disruption. It is obvious that aberrant RAR expression and/or function may play a role in many malignancies (Niederreither and Dollé, 2008; Su and Gudas, 2008; Dollé, 2009; Mark et al., 2009). Tumor growth has thus been linked to RAR overexpression, deletion, mutations, or abnormal post-transcriptional alterations. The RAR-α is over-expressed in several malignancies (Duong and Rochette-Egly, 2011). Its over-expression has been connected to enhance the migration and invasion in several types of carcinomas including head and neck squamous cell carcinoma, pediatric low-grade gliomas, lung adenocarcinoma, and breast carcinoma. In the present study, several *in silico* procedures were used to investigate the RAR-α expression pattern in breast carcinoma. We have further analyzed the effect of the protein docking method to screen a fourth-generation retinoid based therapeutic molecules to target RAR-α.

TIMER 2.0 study results showed that RAR-α is highly expressed in many malignancies including BC, HNSC, KIRC, LUSC, LUAD, LGG, SARC, THCA, and UCEC (Figure 1). A heat map of important RARs and RXRs in breast cancer was created from UCSC Xena. The heat map results also demonstrated that, in comparison to other RARs and RXRs, RAR-α is significantly overexpressed in breast cancer (Figure 2). RAR-α expression analysis in connection to type of sample, age group, subtypes of breast cancer, and ethnicity was further explored using the UALCAN database. The study revealed that RAR-α is greatly expressed in primary tumors than normal patients. The patients with luminal breast cancer displayed increased expression of RAR-α when compared to HER2 or TNBC subtypes. According to the cancer stage, stage 3 showed the highest expression of RAR-α, followed by stage 2, stage 1, and stage 4. Additionally, RAR-α expression was augmented in women between the age of sixty-one to Eighty years old (Figure 3). Moreover, it was found that BC individuals with overexpression of RAR-α had aberrant (WNT, mTOR, and the p53-Rb) signaling pathways (Figure 4).

The bc-GenEXMiner portal was also used to explore the connection between RAR-α and clinicopathological characteristics in breast cancer patients. The findings demonstrated that RAR-α level is typically highly expressed in breast cancer individuals with ER+, PR + receptor expression, nodal negative status, SBR2, and NPI2 grades (Figure 5). The gene-gene interaction of RAR-α was examined using the GeneMANIA portal and the results depicted that RAR-α showed strong interactions with RXR-α, followed by TADA3, MED1, HOXD4, TMSBI10, and several other genes (Figure 6). The PPI of RAR-α was examined by the string database and the results showed a number of genes interacting with RAR-α and network’s top ten hub genes comprised of RARA, RXRA, NCOA1, NCOR1, NCOR2, PPARG, HDAC3, CREBBP, EP300, and SIN3A (Figure 7). Furthermore, the TISCH database revealed that RAR-α is greatly elevated in primary cancers than metastatic cancers. Furthermore, the primary tumor cell population’s expression patterns for RAR-α expression varied greatly and CD4T and CD8T cells expressed higher RAR-α expression in breast cancer patients who had primary tumors (Figure 8).

Using the Enrichr database RAR-α underwent additional analysis for KEGG and gene ontology. Among the biological processes, we analyzed that RAR-α was found to be associated with the positive regulation of T-helper two cell differentiation, IL-13 production, IL-5 production, T-helper cell differentiation, and type 2 immune response. In addition, RAR-α also regulates granulocyte differentiation and has an association with the retinoic acid receptor signaling pathway and cellular response to estrogen stimulus (Figure 9A). RAR-α was mainly abundant in the actin cytoskeleton followed by the cytoskeleton, nucleolus, and nuclear lumen among the cellular compartment (Figure 9B). Among molecular functions, RAR-α was involved in protein kinase B binding, retinoic acid binding, actinin binding, alpha-actinin binding, monocarboxylic acid binding, retinoid binding, transcription coactivator binding, chromatin DNA binding and transcription coregulator binding (Figure 9C). The KEGG pathway study revealed that RAR-α is related with acute myeloid leukemia, Th17 cell differentiation, estrogen signaling pathway, transcriptional mis regulation in cancer including pathways in cancer (Figure 9D).

To find out how RAR-α binds to trifarotene, molecular docking experiments were the next step. Molecular docking studies showed that trifarotene significantly bound to the protein RAR-α, with a binding energy of −8.35 kcal/mol (Figure 10). The stable RMSD plot indicated that the greater ligand affinity of RAR-α + trifarotene contributes to the complex’s stability. Residue changes were seen in the RMSF plot, with significant fluctuations occurring in the residual positions 5–20, 48, 115–125, and 130–148. The Rg decreased from 16.9 to 16.54 Å when the RAR-α Cα-backbone bound to the trifarotene ligand, indicating a highly compact orientation of the protein in the ligand-binding state. Remarkably, while bound with ligand, the SASA value dropped when compared to the unbound state (Figure 11).

Additionally, an *in-vitro* assays was done to confirm our *in silico* study results. Trifarotene showed strong anti-tumor action on MDA-MB-231 and 4T1 BC cell lines, with an IC50 of 9.558 µM and 11.63 µM, respectively, according to the findings of the cell viability MTT test. The low IC50 value indicated that trifarotene significantly inhibits the proliferation of cancer cell (Figure 12).

## 5 Conclusion

According to our research, RAR-α is overexpressed in BC and has carcinogenic features. Treating BC patients with trifarotene that targets RAR-α is an effective treatment approach.

## Data Availability

The BC datasets used in the study are openly assessed as https://portal.gdc.cancer.gov/projects/TCGA-BRCA and the survival data can be accessed as https://kmplot.com/analysis/index.php?p=service&cancer=breast. Also, the databases like ULCAN, GEPIA, and TIMER have used R programming for analysis of datasets.

## Abbreviations

BC: Breast cancer, TISCH: Tumor Immune Single-cell Hub, STRING: Search Tool for the Retrieval of Interacting Genes, RMSD: Root mean square deviation, RAR-α: Retinoic acid receptor alpha, PPI: Protein-Protein interaction, PDB: Protein Data Bank.
